# *In vitro* Evaluation of Antiviral Efficacy of a Standardized Hydroalcoholic Extract of Poplar Type Propolis Against SARS-CoV-2

**DOI:** 10.3389/fmicb.2022.799546

**Published:** 2022-03-08

**Authors:** Giuseppe Sberna, Marco Biagi, Giovanni Marafini, Roberta Nardacci, Mirella Biava, Francesca Colavita, Pierluca Piselli, Elisabetta Miraldi, Gianpiero D’Offizi, Maria Rosaria Capobianchi, Alessandra Amendola

**Affiliations:** ^1^Laboratory of Virology, National Institute for Infectious Diseases INMI, “Lazzaro Spallanzani” Istituto di Ricovero e Cura a Carattere Scientifico (IRCCS), Rome, Italy; ^2^Department of Physical Sciences, Earth and Environment, University of Siena, Siena, Italy; ^3^Clinical Department, National Institute for Infectious Diseases INMI, “Lazzaro Spallanzani” Istituto di Ricovero e Cura a Carattere Scientifico (IRCCS), Rome, Italy; ^4^Epidemiology Department, National Institute for Infectious Diseases INMI, “Lazzaro Spallanzani” Istituto di Ricovero e Cura a Carattere Scientifico (IRCCS), Rome, Italy; ^5^Saint Camillus International University of Health Sciences, Rome, Italy

**Keywords:** SARS-CoV-2, COVID-19, propolis, COVID-19 treatment, pandemic (COVID-19)

## Abstract

Except for specific vaccines and monoclonal antibodies, effective prophylactic or post-exposure therapeutic treatments are currently limited for COVID-19. Propolis, a honeybee’s product, has been suggested as a potential candidate for treatment of COVID-19 for its immunomodulatory properties and for its powerful activity against various types of viruses, including common coronaviruses. However, direct evidence regarding the antiviral activities of this product still remains poorly documented. VERO E6 and CALU3 cell lines were infected with SARS-CoV-2 and cultured in the presence of 12.5 or 25 μg/ml of a standardized Hydroalcoholic Extract acronym (sHEP) of Eurasian poplar type propolis and analyzed for viral RNA transcription, for cell damage by optical and electron microscopy, and for virus infectivity by viral titration at 2, 24, 48, and 72 h post-infection. The three main components of sHEP, caffeic acid phenethyl ester, galangin, and pinocembrin, were tested for the antiviral power, either alone or in combination. On both cell lines, sHEP showed significant effects mainly on CALU3 up to 48 h, i.e., some protection from cytopathic effects and consistent reduction of infected cell number, fewer viral particles inside cellular vesicles, reduction of viral titration in supernatants, dramatic drop of N gene negative sense RNA synthesis, and lower concentration of E gene RNA in cell extracts. Interestingly, pre-treatment of cells with sHEP before virus inoculation induced these same effects described previously and was not able to block virus entry. When used in combination, the three main constituents of sHEP showed antiviral activity at the same levels of sHEP. sHEP has a remarkable ability to hinder the replication of SARS-CoV-2, to limit new cycles of infection, and to protect host cells against the cytopathic effect, albeit with rather variable results. However, sHEP do not block the virus entry into the cells. The antiviral activity observed with the three main components of sHEP used in combination highlights that the mechanism underlying the antiviral activity of sHEP is probably the result of a synergistic effect. These data add further emphasis on the possible therapeutic role of this special honeybee’s product as an adjuvant to official treatments of COVID-19 patients for its direct antiviral activity.

## Introduction

Coronavirus Disease 2019 (COVID-19) has been declared a pandemic on March 11, 2020 by WHO, (2020),^1^ and globally, as of January 25, 2022, there have been 349,641,119 confirmed cases of COVID-19, including 5,592,266 deaths reported in the WHO regions (see text footnote 1).

Although various SARS-CoV-2 specific vaccines are now available with high rate of efficacy, the absence of inhibitors specific for this virus remains a serious problem in counteracting the spread of the infection. Right now, except for remdesivir (Teoh et al., 2020; Kokic et al., 2021), for the monoclonal antibodies (Deb et al., 2021), and for an investigational antiviral molnupiravir indicated for adults with increased risk of progressing to severe COVID-19,^2^ no alternative specific treatment exists for SARS-CoV-2. In fact, although other therapeutic possibilities have been tested, effective compounds have not yet been identified.

Among candidate treatment options for COVID-19, the honeybees’ products (i.e., honey, propolis, bee venom, royal jelly) have been considered an attractive potential therapeutic adjuvant. The significant interest comes from numerous evidence related to the well-known immunoregulatory and anti-inflammatory activities (Sforcin, 2007; Machado et al., 2012; Hori et al., 2013; Governa et al., 2019) and from some experimental data pointing to its therapeutic potential against a variety of viruses such as influenza, HIV, HSV, adenovirus, rotavirus, papilloma virus, and other human coronaviruses (Ito et al., 2001; Shimizu et al., 2008; Maruta, 2014; Huynh et al., 2017).

Propolis is a resinous material collected from bud and exudates of the plants, mixed with bee enzymes, pollen, and wax (Sforcin, 2016; Cornara et al., 2017). Currently, it is widely consumed worldwide as a health aid and immune system stimulator, having been classified as a food or dietary supplement or as a functional/health food (Berretta et al., 2017). Vegetal and geographical origin determines the characteristics of different propolis and many different propolis types are known (Cornara et al., 2017). Brazilian green propolis from *Baccharis* sp. and, mostly, Eurasian poplar type propolis are the two mainly investigated for their use in human health. These propolis show a broad spectrum of properties, thanks to more than 200 bioactive compounds, mainly flavonoids, that exert strong anti-inflammatory, immunomodulatory, and antioxidant activities and antimicrobial, bactericidal, and antiviral power (Zareie, 2011; Shahzad and Cohrs, 2012). Despite numerous *in vitro* and *in vivo* studies, propolis targets and mechanisms of action still remain quite unclear (Sforcin, 2016).

Recently, various components of Eurasian poplar type propolis have been explored for their potential ability to inhibit SARS-CoV-2 infection and replication through molecular docking analysis and *in vitro* and preliminary clinical studies (Berretta et al., 2020; Ali and Kunugi, 2021; Ripari et al., 2021; Silveira et al., 2021).

In particular, molecular modeling of interactions shows that the main propolis molecules that may interfere with SARS-CoV-2 infection are flavonoids and phenolic acids, such as rutin, naringin, caffeic acid phenethyl ester, luteolin, and artepillin C (Ali and Kunugi, 2021). SARS-CoV-2 entry into host cells is characterized by viral spike protein (S) interaction with cellular angiotensin-converting enzyme 2 (ACE2) and transmembrane serine protease 2 (TMPRSS2) (Hoffmann et al., 2020): at least 8 flavonoids (chrysin, galangin, myricetin, rutin, hesperetin, pinocembrin, luteolin, and quercetin) caffeic acid phenethyl ester (CAPE) and caffeic acid in propolis show high binding energy to ACE2 receptor, thus suggesting a marked hampering activity of viral entry (Guler et al., 2021). Another component of propolis, naringin, is able to inhibit *in vitro* the infection of VERO E6 cells with SARS-CoV-2 and to prevent the cytopathic effects (CPE) induced by the virus (Clementi et al., 2021). Furthermore, *in vitro*, rutin, the propolis ethanolic extracts, and the propolis liposomes show the ability to block also all non-structural proteins of SARS-CoV-2 (Refaat et al., 2021). Instead, caffeic acid phenyl ester and withanone are predicted to interact with the main protease Mpro of SARS-CoV-2 and cellular coreceptor TMPRSS2 (Kumar et al., 2020, 2021). In addition, many flavonoids detected in propolis showed high binding affinity toward the RNA-dependent RNA polymerase (RdRp), a crucial enzyme for SARS-CoV-2 replication, with binding scores higher or similar than the reference remdesivir (Elwakil et al., 2021).

Moreover, propolis components were demonstrated to have inhibitory effects also on the cellular PAK1 signaling pathway, whose activation mediates coronavirus-induced lung inflammation, fibrosis, and suppression of adaptive immune response (Maruta, 2014). In fact, in preclinical trials, suppressing PAK1 signaling pathway with propolis administration was effective as anti-SARS-CoV-2 treatment by reducing the viral infection process and pro-inflammatory cytokine release, including IL-6, IL-1 beta, and TNF-α (Berretta et al., 2020), by reducing the hyperactivation of monocytes and macrophages, as well as Jak2/STAT3, NF-κB, and the inflammasome pathways (Cornara et al., 2017; Governa et al., 2019; Berretta et al., 2020; Elmahallawy et al., 2021; Fiorini et al., 2021), and by limiting the risk of cytokine the storm syndrome, the major mortality factor in advanced COVID-19 disease (Berretta et al., 2020; Maruta and He, 2020; Lima et al., 2021).

Because of its antiviral potentials proven in previous *in vitro* and preclinical studies, propolis has been proposed by several parties as a possible prophylactic or adjuvant treatment for managing and treating COVID-19 patients (Bachevski et al., 2020; Maruta and He, 2020; Al Naggar et al., 2021; Ali and Kunugi, 2021; Elmahallawy et al., 2021; Fiorini et al., 2021), and to date, preliminary results seem to confirm these benefits, so further studies and trials are currently ongoing around the world (Berretta et al., 2020; Miryan et al., 2020; Scorza et al., 2020; Al Naggar et al., 2021; Elwakil et al., 2021; Lima et al., 2021; Ripari et al., 2021; Silveira et al., 2021). However, as large differences in chemical composition of different propolis preparations may lead to misleading and variable findings, in the modern pharmacology it is mandatory for the use of propolis products to be standardized, or at least chemically well characterized (Governa et al., 2019; Zaccaria et al., 2019). Similarly, studies that aimed to evaluate the direct antiviral power of propolis by *in vitro* analyses should be performed with preparations of propolis with known and standardized composition because, to date, there are still too few investigations conducted with these standardized formulas.

For this purpose, our study evaluated the ability of a standardized hydroalcoholic extract of poplar type propolis (sHEP) to inhibit SARS-CoV-2 infection *in vitro*, by analyzing the viral transcription by RT-PCR, the infectivity by viral titration, and the CPE by optical and electron microscopy. CAPE, galangin, and pinocembrin, the three main components of the sHEP here used, were also tested for their assumed antiviral power, either alone or in combination.

## Materials and Methods

### Standardized Hydroalcoholic Propolis Extract Preparation and Chemical Characterization

Standardized hydroalcoholic extract of poplar type propolis (sHEP) was prepared by optimizing the method described by Governa et al. (2019). Briefly, the raw material was furnished by Selerbe (Barberino-Tavarnelle Val di Pesa, Firenze, Italy), with quality certificates for pesticides, antibiotics, and aflatoxins. Propolis was extracted in ethanol 80% *v*/*v*, using an ultrasound bath for 3 h.

The solution was filtered, and dry extract was obtained by evaporating the extraction solvent. To verify the chemical composition of sHEP, the total flavonoid quantification and the analysis of its main constituents were performed. Total flavonoids were analyzed by diluting 1:2,000 sHEP (400 mg/ml ethanol 80% *v*/*v*) and were quantified by reading absorbance at 353 nm and interpolating data according to the calibration curve made up with galangin (reference standard grade; Merck/Sigma-Aldrich, Milan, Italy) 78–5,000 mg/L, *R*^2^ = 0.98 (Biagi et al., 2014).

sHEP was also analyzed by HPLC-DAD slightly modifying the procedure of Governa et al. (2019), by using a Shimadzu Prominence LC 2030 3D instrument equipped with a Bondapak C18 column, 10 μm, 125 Å, 3.9 mm × 300 mm column (Waters Corporation, Milford, MA, United States). Water + 0.1% *v*/*v* formic acid (A) and methanol + 0.1% *v*/*v* formic acid (B) were used as mobile phases. The following method was set: A, from 40% at 0 min to 15% after 18 min and then to 40% at 20 min; flow rate was set at 0.8 ml/min. Chromatogram was recorded at 280 nm. Analyses were performed using 10 μl of 1:100 sHEP solution (400 mg/ml ethanol 80% *v*/*v*). CAPE, galangin (GAL), and pinocembrin (PIN) reference grade were purchased from Merck/Sigma-Aldrich (Milan, Italy) and used as external standards. Calibration curves were established using reference standards ranging from 0.008 to 0.500 mg/ml. The correlation coefficient (*R*^2^) of each curve was > 0.99. All chemical analyses were performed in triplicate.

### Cell Culture and Infection Protocol

VERO E6 cells (ATCC Number CRL-1586) and CALU3 (ATCC HTB-55) cell lines were cultivated and maintained in Modified Eagle Medium (MEM; Gibco, Waltham, MA, United States) supplemented with 10% heat-inactivated fetal calf serum (FCS) at 37°C in a humidified atmosphere of 5% CO_2_. They were inoculated with the 2019-nCoV/Italy-INMI1 isolate (Capobianchi et al., 2020) (GenBank accession no. MT008022), here SARS-CoV-2 INMI1, or with SARS-CoV-2 Delta variant, the INMI-648 (GISAD accession no. EPI_ISL_3230211), here vDelta, at a multiplicity of infection (MOI) of 0.001 (or MOI 0.01 when indicated) for 1 h at 37°C and, after two washes, re-suspended in complete medium (at 0.5 × 10^6^ cells/ml) with or without sHEP at indicated concentrations (or individual components at the concentration in which they were present in sHEP) and analyzed after 2, 24, 48, and 72 h post-infection (p.i.) for measurement of various parameters.

Virus titration of isolates was performed on VERO E6 cell line by limiting dilution assay; the viral titer was calculated using the method of Reed and Muench and expressed as tissue culture infectious dose (TCID_50_/ml).

All experiments entailing live SARS-CoV-2 followed the approved standard operating procedures of our biosafety level 3 facility.

### Cell Viability Assay and Imaging of Cytopathic Effects

Cell viability was assessed with the CellTiter-Glo Luminescent Cell Viability Assay (Promega, Madison, WI, United States). CellTiter-Glo Reagent was added directly to the wells at a ratio of 1:1 (volume) with culture supernatants (SNs) and incubated with this mixture at room temperature for 10 min. The luminescence signal was then measured with the Synergy HTX Multi-Mode Reader (BioTek Instruments, Winooski, VT, United States), converted as a percentage with control cells as reference. Images of CPE were obtained at 48 h post-treatment with a Nikon Eclipse Ts2R-FL inverted microscope (Nikon, Konan, Minato-ku, Tokyo, Japan).

### Transmission Electron Microscopy

Transmission electron microscopy (TEM) analysis was performed on VERO E6 cells using standard procedures. Cells at different conditions of treatment were collected 24 h p.i. and fixed with 2.5% glutaraldehyde in 0.1 M cacodylate buffer, for 30 min at 4°C. Post-fixation was performed with 1% OsO_4_. Samples were then dehydrated in graded ethanol and embedded in Epon resin. Ultrathin sections were stained with 2% uranyl acetate and observed under a transmission electron microscope JEOL JEM 2100 Plus (Japan Electron Optics Laboratory Co., Ltd., Tokyo, Japan). Images were captured digitally with a TVIPS digital camera (Tietz Video and Image Processing Systems GmbH, Gauting, Germany). The percentage of SARS-CoV-2–infected VERO E6 cells was evaluated by analyzing at least 40 cells per condition at the electron microscope. Cell counting was done by two independent researchers; data are presented as mean ± SD.

### Detection of Viral RNA Transcripts in Cell Extracts and in Supernatants

Total viral RNA was extracted from VERO E6 and CALU3 cellular pellet using Trizol (Life Technologies, New York, NY, United States) and from their SNs with QIAamp Viral RNA Mini Kit (Qiagen) system, according to the manufacturer’s instructions. In cellular extracts, SARS-CoV-2 RNA was amplified by real-time quantitative RT-PCR (qRT-PCR) in a Rotor-GeneQ Real-Time cycler (Qiagen, Hilden, Germany) with the RealStar SARS-CoV-2 RT-PCR Kit (Altona Diagnostics, Hamburg, Germany) that amplifies the E gene specific for Betacoronaviruses lineage B and the S gene for SARS-CoV-2. Since the cycle threshold (Ct) values of the two amplified genes (E and S genes) were superimposable, for convenience, in graphs were shown only the results related to the E gene. To measure negative sense viral RNA of N gene in cellular extracts, the reverse transcription step was minus strand-specific, based on the use of the N gene forward primer only, as described for the detection of other viral RNAs (Biava et al., 2017). After treatment with 1 μl of RNase H (20 U/μl) for 20 min at 37°C, cDNA was amplified with SuperScript III One-Step RT-PCR System kit (Invitrogen, Karlsruhe, Germany) with a 25-μl reaction mixture under the following conditions: 0.5 μl of kit enzyme mixture, 12.5 μl of 2 × Reaction Mix, 0.8 μl of MgSO_4_, 0.5 μl of 25 μM primer mix, 0.5 μl of 20 μM of probe, 4.7 μl of nuclease free water (Mol Biograde, Hamburg, Germany) and 5 μl of cDNA. The following modified thermal profile, omitting the reverse transcription step, was used: 2 min at 95°C for reverse transcriptase inactivation and DNA polymerase activation followed by 45 amplification cycles of 15 s at 95°C and 1 min at 60°C. Primers and probe sequences are described elsewhere (Corman et al., 2020). Normalization of Ct values was performed using a housekeeping gene (RNaseP) in qRT-PCR according to the CDC protocol of real-time RT-PCR for influenza A (H1N1).

The Simplexa COVID-19 Direct Assay (Diasorin Molecular, Saluggia, Italy) was used to monitor the viral yield in the supernatant of the two cell lines. This assay is a real-time RT-PCR system that enables the direct amplification of SARS-CoV-2 RNA without sample processing like RNA extraction. Two different regions of the SARS-CoV-2 genome were amplified: ORF1ab and S gene; an RNA internal control was used to detect RT-PCR failure and/or inhibition. The Simplexa COVID-19 direct assay was used according to the manufacturer’s instructions. Since the Ct values of the two amplified genes (ORF1ab and S genes) were superimposable, for convenience, in graphs were shown only the results related to ORF1ab gene.

### Immunofluorescence

After the infection with SARS-CoV-2 isolates, cells were fixed for 15 min with 4% paraformaldehyde, permeabilized with PBS containing Tween 20 (Merck Life Science S.r.l.) and incubated with COVID-19 patient–derived serum at 4°C for 1 h. Cells were then washed twice with PBS and Tween 20 and incubated in the dark for 30 min at RT with FITC-conjugated goat anti-human IgG antibodies (Euroimmun, DE). Images were obtained using Nikon Eclipse E600 microscope equipped with NIS-Elements D5.21.00.

### Statistical Analysis

Data management, analyses, and graphs were performed using GraphPad Prism version 8.00 (GraphPad Software, La Jolla, CA, United States) and Excel 2016 (Microsoft Office). The Student *t*-test, Pearson coefficient correlation, and linear regression analysis were performed; statistical significance was set at *p*-value < 0.05. Samples with Ct values < 40 were considered positive. For statistical calculations, an arbitrary value of 40.01 Ct was assigned to all negative samples (i.e., those with Ct > 40.01).

## Results

### Chemical Composition of sHEP

Chemical composition of sHEP is summarized in Table 1. The sHEP here used resulted almost identical to that investigated by Governa et al. (2019) and confirmed to have the quali-quantitative chemical profile required for a very high-quality poplar type propolis (Gardana et al., 2007).

**TABLE 1:** Chemical composition of sHEP.

Constituent	Content (mg/g propolis ± SD)
Total flavonoids (expressed as galangin)	155.01 (±18.16)
Galangin	42.69 (±0.30)
Pinocembrin	26.59 (±0.21)
Caffeic acid phenylethyl ester	10.51 (±0.29)

### Cell Lines and Assessment of Viability in Presence of sHEP

VERO E6 (African green monkey, kidney epithelial cell line) and CALU3 (human lung epithelial cell lines) are both permissive to SARS-CoV-2 infection and productive for viral replication (Chu et al., 2020); however, CALU3 differs from VERO E6 for the expression of TMPRSS2 receptor and production of IFN-γ (Yamamoto et al., 2016). Cell viability of VERO E6 and CALU 3 was assessed up to 72 h of culture in presence of nine different concentrations of the sHEP (400–200–100–50–25–12.5–6–3–1 μg/ml) for measuring the cytotoxic concentration 50% (CC_50_). At each time point (2, 24, 48, and 72 h of culture), the measured CC_50_ values were 189, 165, 99, and 83 μg/ml, respectively, for VERO E6 (Supplementary Figure 1A). For CALU 3, CC_50_ values were 181, 164, 113, and 55 μg/ml of sHEP, respectively (Supplementary Figure 1E). Based on these data, it was decided to use a dose of sHEP lower to the CC_50_ value, which had been well tolerated by both cell lines during the experiments, namely 25 μg/ml of sHEP. In addition, doses of 12.5 and 50 μg/ml sHEP were also used as controls in many experiments.

During culturing in presence of increasing concentrations of sHEP up to 25 μg/ml, a progressive reduction of cell growth directly related with sHEP dose was observed, however without CPE, in line with the evidence that water extracts of propolis inhibit different cell line growth with cytostatic effects (Najafi et al., 2007). Indeed, up to 48 h of culture in presence of 25 μg/ml sHEP, no alteration of cell morphology was observed (Supplementary Figures 1B–D,F–H). Curiously, CALU3 acquired a triangular-like and elongated shape, yet without visible cytotoxic effects (Supplementary Figure 1H).

### sHEP Protected VERO E6 and CALU3 From Cytopathic Effects Induced by SARS-CoV-2 Infection

**FIGURE 1:**
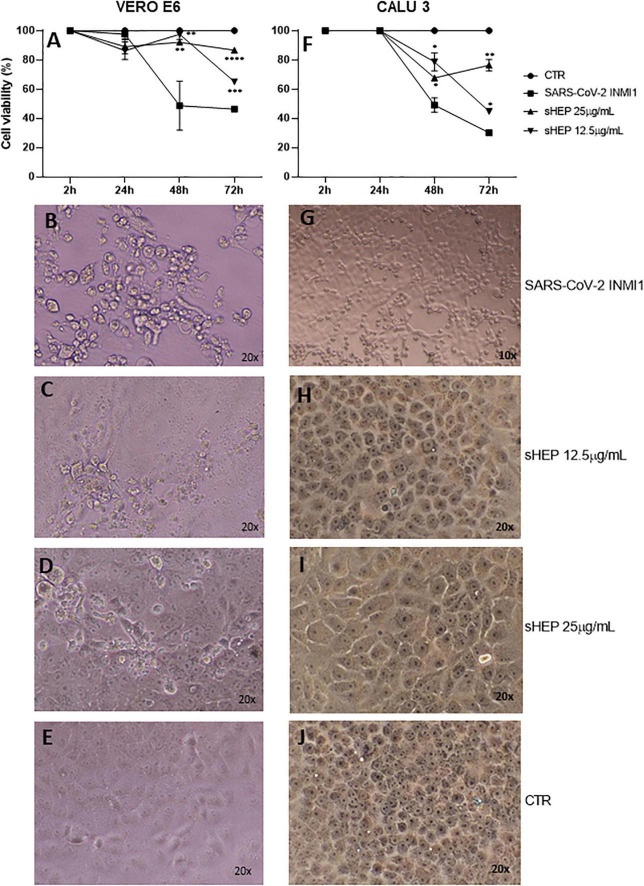
Effects of sHEP addition in the medium of culture of SARS-CoV-2 infected VERO E6 and CALU3. Panels **(A,F)** VERO E6 and CALU3 cell viability was measured with CellTiter-Glo ^®^ Luminescent Cell Viability Assay. After SARS-CoV-2 infection (MOI0.001) for l h at 37^°^C and sHEP addition in the culture medium, cell viability was assessed at each indicated time points and compared with that of control infected cells. Data points represent the mean (+SD) of three independent experiments. Panels **(B–D)** and panels **(G–I)** show light microscopy images of VERO E6 and CALU 3 cell lines, respectively, 48 h after SARS-CoV-2 infection and culture in presence of sHEP at indicated concentrations. Panels **(E,J)** refer to control uninfected cells. The asterisks indicate statistically significant differences determined by Student’s *t*-test (****p < 0.0001; ***p < 0.001; **p < 0.01; *p < 0.05; no asterisk, *p* ≥ 0.05) between infected cell and those infected and cultured in presence of sHEP.

When the two cell lines were infected with SARS-CoV-2 INMI1 isolate, the addition of sHEP in medium culture, immediately administered after the two washes to eliminate the virus in excess, was able to protect the cells against the CPE induced by the virus in the following hours (Figure 1). Cell viability of both cell lines infected with SARS-CoV-2 INMI1 isolate and cultured in medium alone or in presence of 12.5 μg/ml and 25 μg/ml sHEP is shown in Figures 1A,F. Compared with infected cells, those growing in presence of sHEP maintained significantly higher percentage of viability with both concentrations of sHEP and throughout the experiment, and CPE were not appreciable. In particular, in presence of 25 μg/ml sHEP, the cell viability percentage of infected VERO E6 remained above 80% until 72 h p.i. (91.8% at 48 h p.i., *p* = 0.0034 vs. SARS-CoV-2 and 86.0% at 72 h p.i., *p* < 0.0001 vs. SARS-CoV-2); in CALU3, it was maintained around 70% (67.6% at 48 h p.i., *p* = 0.0339 vs. SARS-CoV-2 and 76.4% at 72 h p.i., *p* = 0.0039 vs. SARS-CoV-2). By contrast, in absence of sHEP, cell viability of infected cells dropped below 50% or more at 48 h and 72 h p.i. in both cell lines due to the CPE resulting from viral infection. Interestingly, images captured at optical microscopy showed that while in VERO E6 sHEP reduced CPE in a significant quote of cells, in CALU3 sHEP was capable to prevent cell damage almost completely [compare the images of infected cells shown in Figure 1B *vs*. those of VERO E6 treated with 12.5 μg/ml and 25 μg/ml sHEP in panels (C), (D), and (G) vs. those of CALU3 in panels (H) and (I)].

### Electron Microscopy Features of SARS-CoV-2–Infected Cells Treated With sHEP

**FIGURE 2:**
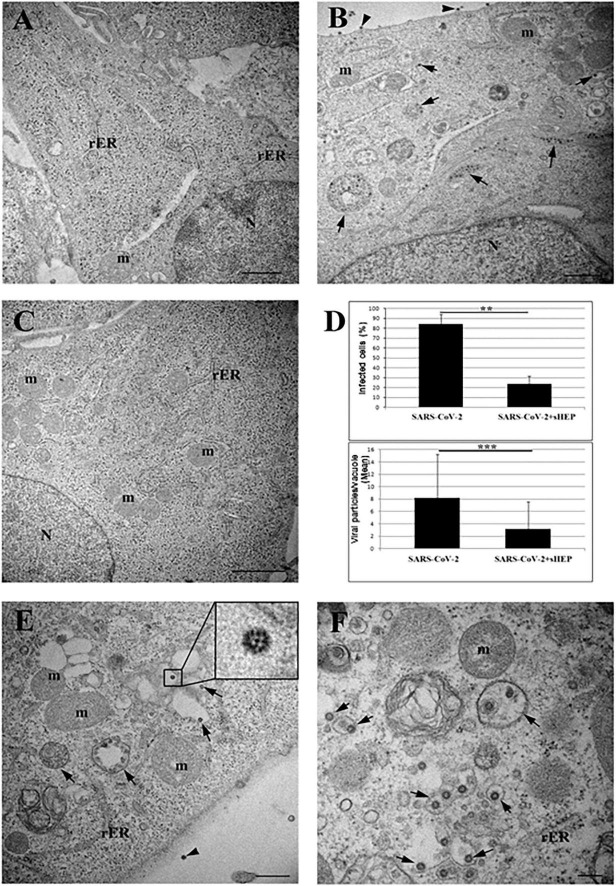
Electron microscopy ultrastructure features of SARS-CoV-2-hfected VERO E6 cells treated with sHEP. Panel **(A)** the image shows the typical morphology of VERO E6 cells after 24 h culturing in presence of sHEP 25 μg/mL. No signs of ultrastructural alterations were evident. Panel **(B)** SARS-CoV-2 infected VERO E6 cell: arrows point to membrane bound vacuoles containing numerous viral particles. Mature viral particles are visible at the cell surface (arrowheads). Panel **(C)** among infected VERO E6 cultured in presence of sHEP 25 μg/mL, numerous uninfected cells were observed showing normal intracellular morphology and no sign of viral presence, as here shown. Panel **(D)** percentage of infected cells (top graph) and mean number of viral particles counted within vesicles (bottom graph) measured in infected cultures were compared to those observed among cultures infected and treated with 25 μg/mL sHEP (SARS-CoV-2 + sHEP). The differences were significant. Data reported as means values ± SD (**p < 0.01; ***p < 0.001; no asterisk, *p* ≥ 0.05). Panel **(E)** sHEP 25 μg/mL treated SARS-CoV-2 infected cell. Arrows point to membrane bound vacuoles containing typical viral particles. Mature viral particle is visible at the cell surface (arrowhead). Higher magnification of viral particle is visible in the boxed area: black dots are visible inside the viral particles due to cross section through the nucleocapside. Panel **(F)** higher magnification of an infected cell showing numerous single virions, or small group of virions, enclosed in single membrane vacuoles (arrows). N, nucleus; m, mitochondrion; rER, rough endoplasmic reticulum. Scale bars: *A,B* = l μm; *C* = 2 μm; *E* = 500 nm; *F* = 200 nm.

To analyze the effects of sHEP at ultrastructural level, we examined VERO E6 cells infected with SARS-CoV-2 and cultured in presence of sHEP 25 μg/ml after 24 h p.i. with TEM. As shown in Figure 2A, sHEP alone does not induce evident ultrastructural alterations. Cells infected with SARS-CoV-2 showed mature viral particles at the cell surface and vacuoles containing numerous viral particles in the cytoplasm (Figure 2B), as previously observed (Eymieux et al., 2021; Nardacci et al., 2021). Among the SARS-CoV-2–infected VERO E6 treated with sHEP, instead, numerous cells without signs of viral infection and normal intracellular morphology were observed (Figure 2C) and those infected were significantly lower in percentage compared with those infected and cultured in absence of sHEP [*p* = 0.01 vs. positive control (i.e., SARS-CoV-2); Figure 2D (top)]. Furthermore, these last cells showed numerous single virions, or small group of virions, enclosed in single membrane vacuoles [Figure 2, panels (D, bottom), (E), and (F)], differently from untreated infected cells (Figure 2B).

### Effects of sHEP on SARS-CoV-2 Replication: Analysis in Cellular Extracts of Viral RNA Transcripts

**FIGURE 3:**
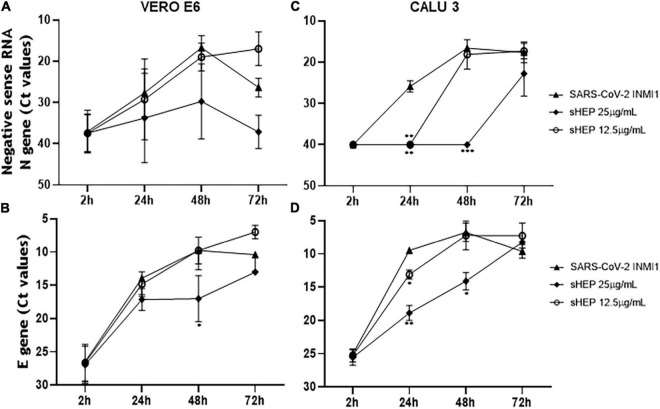
Kinetics of intracellular SARS-CoV-2 N and E genes expression in VERO E6 and CALU3. After viral infection, sHEP was added at 12.5 or 25 μg/mL and SARS-CoV-2 N and E genes RNA transcripts expression was evaluated at indicated time points by real-time RT-PCR. Panels **(A,C)** Ct values refer to negative sense RNA transcripts for N gene detected in VERO E6 and CALU3 cell extracts, respectively. Panel **(A)** in VERO E6, no significant differences were observed. Panel **(C)** in CALU3, the negative sense RNA transcript levels of the N gene were significantly lower at 24 h p.i. (*p* = 0.0024) and at 48 h p.i. (*p* = 0.0002) with 25 μg/mL sHEP, with respect to untreated but infected cells. Panels **(B,D)** Ct values of E gene RNA detected in VERO E6 and CALU3 cell extracts, respectively. Panel **(B)** in VERO E6, E gene was significantly lower at 48 h p.i. (*p* = 0.05) Panel **(D)** accordingly, E gene RNA transcripts in CALU3 were also significantly lower at 24 h and 48 h p.i. (*p* = 0.066, and *p* = 0.0302, respectively). Data points represent the mean (±SD) of three independent experiments, each performed in quadruplicate. The asterisks indicate statistically significant differences determined by Student’s *t*-test (***p < 0.001, **p < 0.01; *p < 0.05; no asterisk, *p* ≥ 0.05) between infected cells and those infected and cultured in presence of sHEP.

The power to hinder viral replication by sHEP was analyzed by following over time the levels of the newly synthetized negative sense RNA transcripts of N gene from the viral genome. At each time points, both SNs and cell extracts were harvested and analyzed by real-time RT-PCR to measure levels of negative sense transcripts (Figures 3A,C). The Ct values for these transcripts were higher (meaning lower levels of viral load) when both the infected cell lines were cultured in presence of sHEP, mainly at 25 μg/ml, thus pointing out the ability of sHEP to exert its antiviral effect directly at intracellular level. Although no significant differences were observed for VERO E6 (Figure 3A), in CALU3 the negative sense RNA transcript levels of the N gene were significantly lower at 24 h p.i. (*p* = 0.0024) and at 48 h p.i. (*p* = 0.0002) with 25 μg/ml sHEP and at 24 h p.i. (*p* = 0.0043) also with 12.5 μg/ml sHEP, compared with untreated but infected cells (Figure 3C). This effect was confirmed by measuring the positive sense RNA transcripts of the viral E gene in the same cell extracts (Figures 3B,D). The remarkable effectiveness of sHEP in limiting the synthesis of new viral transcripts was much more noticeable in CALU3 already at 24 h (Figures 3C,D).

### Effects of sHEP on SARS-CoV-2 Yield: Viral Titration and Genomic Detection in Supernatants

**FIGURE 4:**
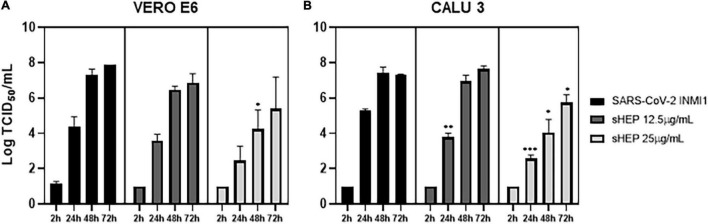
Effects of sHEP on SAR-CoV-2 yield in SNs of VERO E6 and CALU3 cell lines. Panels **(A,B)** SARS-CoV-2 titration (Log TCID_50_/mL) in SNs of VERO E6 and CALU3, respectively, after infection and culturing in presence of 12.5 or 25 μg/mL sHEP or medium alone was measured at indicated time points. Data represent the mean (+SD) of three independent experiments. Virus titration was performed on VERO E6 cell line by limiting dilution assay; the viral titer was calculated using the method of Reed and Muench and expressed as tissue culture infectious dose TCID_50_/mL. The asterisks indicate statistically significant differences determined by Student’s *t*-test (***, *p* < 0.001, **, *p* < 0.01; *p < 0.05; no asterisk, *p* ≥ 0.05) between Infected cells and those infected and cultured in presence of sHEP.

The infectivity of viral particles yielded in SNs during treatment with sHEP was assessed by viral titration test, aimed to determinate the 50% tissue culture infective dose (TCID_50_/ml). SNs of both infected and sHEP-treated cell lines were collected at indicated time points and exposed to uninfected VERO E6 for 72 h. As shown in Figure 4A, viral titer measured in SNs collected from VERO E6 treated with sHEP was lower compared with infected cells untreated with sHEP, with a significant difference appreciated with 25 μg/ml sHEP at 48 h p.i. (*p* = 0.049 vs. SARS-CoV-2).

However, with CALU3, viral titer in SNs from cultures carried out in presence of 25 μg/ml sHEP was significantly reduced with a dose- and time-related kinetics, when compared with titers measured in SNs of infected with the virus alone at all time points considered (24 h p.i.: *p* = 0.0002; 48 h p.i.: *p* = 0.0134; 72 h p.i. *p* = 0.0222 *vs.* SARS-CoV-2), in accordance with the data presented previously (Figure 3). Interestingly, sHEP 12.5 μg/ml was also able to significantly hamper viral release in SNs at 24 h p.i., compared with untreated infected cells (*p* = 0.0036 *vs.* SARS-CoV-2), but this concentration became ineffective in the following hours (Figure 4B).

In perfect agreement with these results were the ORF1ab positive sense RNA levels detected by real-time RT-PCR in these same SNs. In VERO E6, the Ct values of ORF1ab detected in SNs of infected cells cultured with sHEP were slightly higher (meaning lower viral load) from those of infected cultures carried out in absence of sHEP; in CALU3, the hindering effect exerted by sHEP induced significantly lower viral loads (higher Ct values) at 24, 48, and 72 h p.i. The percentages of viral yield inhibition in SNs induced by sHEP in both cell lines are shown in Table 2.

**TABLE 2:** Viral yield inhibition in SNs (the viral yield inhibition in SNs was induced by adding sHEP in culture medium after virus inoculation was calculated from ORF1ab gene Ct values).

Viral yield inhibition in SN (%) ± SD	VERO E6				CALU3			
	2 h	24 h	48 h	72 h	2 h	24 h	48 h	72 h
sHEP 12.5 μg/ml	5.0 (±4.5)	14.7 (±10.8)	−2.2 (±6.7)	1.7 (±0.07)	−6.5 (±8.4)	20.4 (±1.6)	6.7 (±5.8)	−0.8 (±1.2)
sHEP 25 μg/ml	0.0 (±1.5)	30.7 (±15.0)	21.1 (±17.1)	24.8 (±28.4)	4.3 (±2.7)	42.6 (±2.1)	45.8 (±9.9)	23.0 (±12.3)

### Assessment of the Antiviral Activity by Single Components of sHEP

**FIGURE 5:**
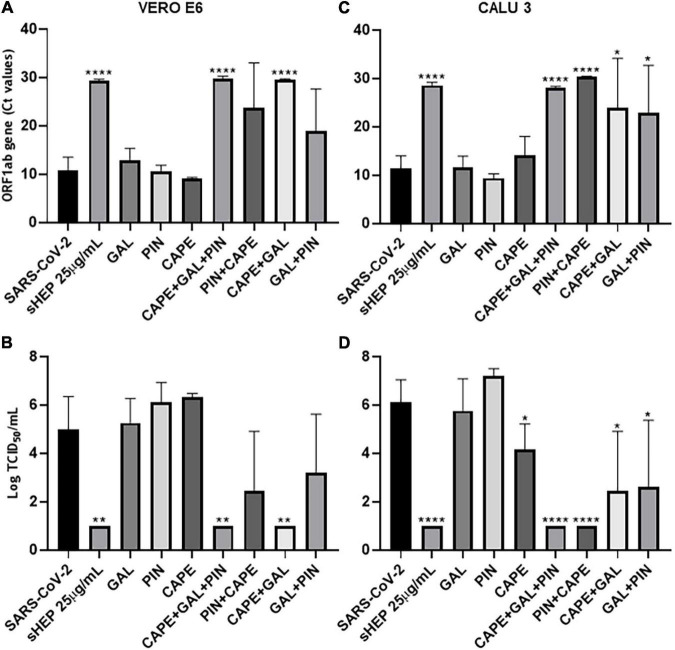
Ability of the three main components of sHEP, the Caffeic Acid Phenethyl Ester (CAPE), the Galangin (GAL), and the Pinocembrin (PIN), to reduce viral replication on infected VERO E6 and CALU 3 cells. The three components of sHEP where added to the culture medium of VERO E6 and CALU 3 after virus inoculation, at the same concentration they were contained in sHEP. The sHEP components were tested singly (CAPE or GAL or PIN), or in combination two by two (CAPE + GAL; GAL + PIN; CAPE + PIN), or all together (CAPE + GAL + PIN), as indicated in the graphs; as a reference, the values measured in the presence of sHEP (sHEP 25 μg/mL) or the SARS-CoV-2 INMI1 alone (SARS-CoV-2) were also shown. After 48 h of culture, viral yield and virus titration in SNs were analyzed. Panels **(A,C)**: viral yield (expressed as Ct values of ORF1ab gene transcripts) observed in SNs of VERO E6 and CALU3, respectively. Panels **(B,D)**: virus titration (expressed as Tissue Culture Infectious Dose TCID_50_/mL) measured in SNs of VERO E6 and CALU3, respectively. Data represent the mean (±SD) of three independent experiments. The asterisks indicate statistically significant differences determined by Student’s *t*-test (****p < 0.0001, **p < 0.01; *p < 0.05; no asterisk, *p* ≥ 0.05) between infected cells and those infected and cultured in presence of sHEP or components used singly or in combination.

The main components of sHEP, CAPE, GAL, and PIN, were assessed separately, or in combination two by two, or altogether for their ability to reduce viral release in SNs of culture at 48 h p.i. The three single molecules were added to the culture medium at the same concentrations they are in sHEP. ORF1ab RNA levels and viral titration were assessed in SNs at the indicated time points (Figure 5). The three components were not effective in inhibiting viral replication when added one by one to the culture medium in both cell lines (Figure 5A for VERO E6 and Figure 5B for CALU3). Conversely, the combination of the three molecules (CAPE + GAL + PIN) was extremely effective (VERO E6: *p* = 0.0002 *vs.* SARS-CoV-2; CALU3 *p* = 0.009 *vs*. SARS-CoV-2), so much to limit viral replication to the same levels as those observed with sHEP, thus demonstrating that the antiviral effect of sHEP is mainly due to a synergistic effect of the main characteristic polyphenols of poplar type propolis. The same components added two by two showed different efficacy, depending on the combination and cell line. In fact, CAPE was efficient when combined with GAL in VERO E6 cells, while in CALU3 showed higher antiviral power associated with PIN. These different results deserve to be further explored.

### No Effects of sHEP in Preventing SARS-CoV-2 Entry Into the Cells

To evaluate the ability of sHEP to inhibit virus entry into cells, the two cell lines were pre-incubated for 1 h with 12.5 and 25 μg/ml sHEP, then inoculated with the virus and, after two washes, cultured in the presence of sHEP at the same dose used in the pre-incubation. At the same time points, cell viability, N gene negative sense RNA, E gene transcripts, and viral titration were assessed in both cell lines (Supplementary Figure 2). This treatment produced results very similar to those obtained by adding sHEP only after viral inoculation and, again, more significant viral inhibition values were observed with CALU3 (Supplementary Figure 2). For example, for N gene negative sense RNA in CALU3 at 2, 24, 48, and 72 h p.i., the *p*-values were *p* > 0.05, *p* = 0.0043, *p* = 0.0003, and *p* > 0.05, respectively, compared with sHEP-untreated cells. Furthermore, viral titration of SNs from CALU3 at 2, 24, 48, and 72 h p.i. showed significant different *p*-values, which were *p* > 0.05, *p* < 0.0001, *p* = 0.0036, and *p* = 0.0139, respectively. Altogether, these data indicate that the antiviral power of sHEP is carried out essentially downstream of SARS-CoV-2 infection.

### Immunostaining of SARS-CoV-2–Infected Cells

**FIGURE 6:**
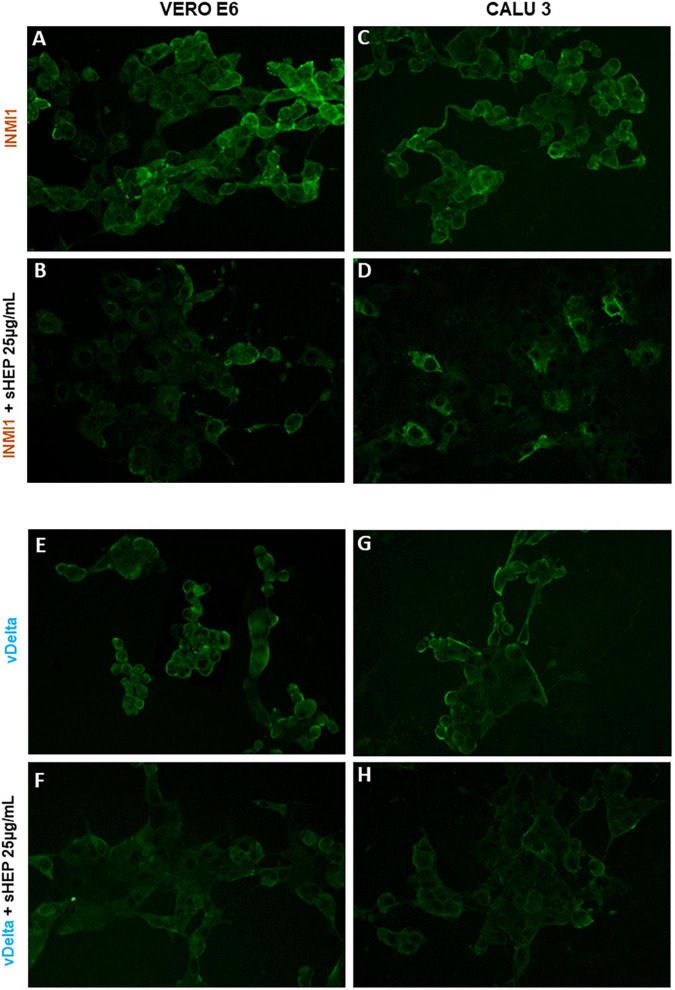
Immunofluorescence staining for SARS-CoV-2 of VERO E6 and GALU 3 cells 48 h post-infection with INMI1 or vDelta and treated with sHEP. Panels **(A,C)**: VERO E6 and CALU 3 infected with INMI1, respectively; Panels **(B**,**D)** show these same cells respectively, infected with INMI1 and incubated with sHEP 25 μg/mL for 48 h. Panels **(E**,**G)** refer to VERO E6 and CALU 3 after 48 h infection with vDelta; Panels **(F**,**H)** show the same cells respectively, infected with vDelta and treated with sHEP 25 μg/mL. The staining with human anti-SARS-CoV-2 antibodies illustrates the difference in fluorescence intensity between cells infected and those infected and then incubated in presence of sHEP. Magnification: 20X.

The ability of sHEP to limit viral replication was confirmed by immunofluorescence. VERO E6 and CALU3 infected with SARS-CoV-2 INMI1 or vDelta were cultured in presence or not of sHEP 25 μg/ml and subjected to immunofluorescence with human serum obtained from COVID-19 patients after 48 h of culture. As shown in Figure 6, cells infected with SARS-CoV-2 [INMI1: panels (A) and (C); vDelta: panels (E) and (G)] showed strong fluorescence intensity related to considerable presence of the virus. By contrast, among cells infected and cultured with 25 μg/ml sHEP, lower fluorescence intensity was observed, compared with infected and cultured in medium alone.

### Effect of sHEP at Higher Multiplicity of Infection

When the two cell lines were infected with SARS-CoV-2 INMI1 at higher MOI 0.01 and cultured with sHEP, the dose of sHEP necessary to maintain the cell viability around 80% at 48 h p.i. was again 25 μg/ml (Supplementary Figures 3A,C). However, sHEP addition failed to limit the virus release in SNs in both cell lines (Supplementary Figures 3B,D).

## Discussion

Currently, in the fight against the SARS-CoV-2 pandemic, only vaccinations are proving full effectiveness against the virus (Pritchard et al., 2021). Indeed, remdesivir demonstrated, with a moderate quality of evidence, no benefit in mortality rate and, with low or very low quality of evidence, benefits in terms of rates of clinical improvement and faster time to clinical improvement (Singh et al., 2021). Moreover, the monoclonal antibodies against SARS-CoV-2, although effective as post-exposure prophylaxis to prevent severe diseases or complications, show several limitations, such as difficulties in development and production, high economic costs, and loss of efficacy against new viral variants. Accordingly, a feverish research of drugs against SARS-CoV-2 is ongoing and natural products have become alternatives to consider being often free of toxic or side effects, cheap, and readily available all over the world (Mani et al., 2020). Propolis is one of the most intriguing products, thanks to the high concentration of bioactive of phenolic acids, flavonoids, and terpenes that exert strong antimicrobial, anti-inflammatory, and immunomodulatory activities, thus reducing the activation of the cytokine storm and the risk of comorbidities that complicate the clinical course of COVID-19 (Sforcin et al., 2000; Machado et al., 2012; Chan et al., 2013; Galeotti et al., 2018; Bachevski et al., 2020; Berretta et al., 2020; Maruta and He, 2020; Scorza et al., 2020; Al Naggar et al., 2021; Ali and Kunugi, 2021; Elmahallawy et al., 2021; Keflie and Biesalski, 2021; Lima et al., 2021; Ripari et al., 2021; Rivera-Yañez et al., 2021; Rodrigues et al., 2021). Consequently, propolis have been proposed as prophylactic or adjuvant for COVID-19 treatment.

Moreover, some of the main constituents of propolis (such as caffeic acid, galangin, and pinocembrin) were predicted also to inhibit both the TMPRSS2 and the ACE2 receptors on host cells, the viral protein Spike, and the viral Mpro and RdRp enzymes, all of them crucial elements for entry and replication of SARS-CoV-2 (Bachevski et al., 2020; Berretta et al., 2020; Dai et al., 2020; Gao et al., 2020; Jin et al., 2020; Kumar et al., 2020, 2021; Maruta and He, 2020).

Based on all these evidences, clinical trials involving the administration of propolis alone or in association with standard care therapies are currently ongoing and substantial clinical benefits in hospitalized COVID-19 patients have been in fact already described. For example, in a randomized, controlled, open-label, single-center trial conducted in Brazil (registered as ClinicalTrials.gov number NCT04480593), hospitalized adult COVID-19 patients were treated with standard care therapies plus an oral dose of 400 mg or 800 mg/day of green propolis extract (EPP-AF) for 7 days. Propolis was safe and beneficial and the length of hospital stay post-intervention was significantly shorter. Patients treated with propolis had a reduced need for invasive oxygen therapy and administration of propolis early in the disease would seem to have even greater benefit in reducing the disease’s consequences (Berretta et al., 2020; Ribeiro et al., 2021; Ripari et al., 2021; Rivera-Yañez et al., 2021; Silveira et al., 2021). Another study reported that hospitalized COVID-19 patients receiving green Brazilian propolis anticipated viral clearance, symptom recovery, and discharge from the hospital. Interestingly, to date, no patient discontinued propolis treatment for adverse events (Silveira et al., 2021), even though allergic reactions against some components of propolis are not an uncommon occurrence, as cases of allergic dermatitis and hypersensitivity reactions have been previously described (Bellegrandi et al., 1996; Kurek-Górecka et al., 2020).

However, it is important to point out that propolis used in ongoing clinical trials consist of new standardized formulations, almost always in the form of extracts, which guarantee chemically and biologically reproducible compositions, high safety levels of purity and effectiveness, and minimum concentrations of toxic substances (Sforcin and Bankova, 2011; Berretta et al., 2012; Silveira et al., 2019; Zaccaria et al., 2019; Osés et al., 2020; Rivera-Yañez et al., 2021). Experimental data showing the effects exerted by the standardized extracts of propolis on viral infection and replication *in vitro* are still very scarce, but they need to be confirmed in depth.

For these reasons, we decided to test and analyze *in vitro* a chemically well-characterized preparation of a poplar propolis extract, with two different cell lines infected with SARS-CoV-2, to ascertain the antiviral effects and to investigate the virological aspects.

We demonstrated that addition of sHEP in culture medium of both cell lines, after their infection with SARS-CoV-2, hampered the viral replication by interfering right at the level of the synthesis of new viral RNA transcripts inside the cells. As a consequence, a significant time- and dose-dependent decrease in the yield of infectious viral particles occurred, confirmed by significant lower viral loads and lower viral titers in supernatants.

Another evidence of the powerful antiviral effect exerted by the sHEP here used was the reduction of CPE induced by the virus, observed already in VERO E6 and even more in CALU3. Further confirmation was the smaller amount of viral particles counted within the cellular vesicles when sHEP was added to the culture medium. The fact that CALU3 have characteristics similar to human lung epithelial cells and that in the presence of sHEP they resisted well to the devastating impact of viral infection further support the hypothesis of antiviral power of this complex and, in parallel, indirectly explain the beneficial effects observed on COVID-19 patients. In fact, another interesting finding that emerged from this study was that sHEP was able to exert its antiviral power essentially downstream of infection, i.e., after virus entry, while pre-treating of cells with sHEP prior to infection with SARS-CoV-2 was unable to interfere with the infection. This observation has important clinical relapses that perfectly fit in and provide a plausible explanation for the beneficial effects registered in ongoing clinical trials, where patients already infected by SARS-CoV-2 show significant clinical improvements after starting treatment with other propolis.

Our data also add another piece of information to the complicated puzzle of propolis-mediated effects. Using the three main components of the standardized mixture of the propolis extract here used (caffeic acid, galangin, and pinocembrin), we observed that they were ineffective if administered individually in infected cell cultures; by contrast, when the three molecules were used in combination, they were able to exercise a similar antiviral power as the whole sHEP, thus confirming that the effects of this mixture are essentially due to a synergistic action mediated by several components present in certain concentrations.

Besides the positive effects of sHEP, inter- and intra-experimental variability of results was nevertheless observed. This can be explained by the fact that propolis extracts appear as poorly soluble mixtures and contain compounds with varying degrees of toxicity, probably even in standardized formula. Therefore, by isolating and identifying the components with effective antiviral properties, it is possible to prepare solutions deprived of toxic or useless components, which could acquire a greater and specific antiviral power and no side effects.

The strength of this study is to have demonstrated the antiviral effects of a standardized preparation of propolis from a deeply virological point of view, with promising results across the board for its use in clinical setting. One limit is the *in vitro* experimental model used, which needs to be translated into a 3D primary culture model of human lung epithelial cells to further elucidate on the antiviral mechanisms triggered into the cells by this precious gift from the world of nature.

Although sHEP appears to be promising in the fight against the new coronavirus, further studies focusing on the identification of the main molecules responsible for the antiviral effects are needed to elucidate targets and mechanisms of action. Furthermore, pharmacokinetic and pharmacodynamic analyses are required for each of the individual components since they have different characteristics from each other. Finally, it will be also necessary to establish which preparation to use, the safety concentrations, and the best methods of administration in infected tissues (i.e., oral intake, inhalation, nasal spray, etc.) to avoid side effects, even though oral doses of 400 mg or 800 mg/day of green propolis extract are already in use in registered official trials. In our opinion, the use of purified components with antiviral power in combination could be the safest method of administration of such product, not only to ensure the lowest rate of adverse events, especially in the most sensitive individuals prone to allergic reactions, but also to ensure selective and specific antiviral effects, and our results confirmed this alternative use of propolis.

## Conclusion

In conclusion, we demonstrated that standardized propolis preparations and their components have the capacity to hinder SARS-CoV-2 infection at various levels. These formulations deserve to be introduced in new lines of experimental and clinical researches to definitively establish their clinical efficacy in prevention of infection and disease progression.

## Data Availability Statement

The raw data supporting the conclusions of this article will be made available by the authors, without undue reservation.

## Author Contributions

GS, AA, MiB, GM, and RN performed the experiments. AA, GS, and MaB conceived the experiments. MaB and FC provided the materials. MC and MaB supervised the experiments. MC provided the funding. AA and GS wrote the manuscript. AA, GS, and PP analyzed the data. MaB, MC, GD’O, and EM critically revised the manuscript. All authors contributed to the article and approved the submitted version.

## Conflict of Interest

The authors declare that the research was conducted in the absence of any commercial or financial relationships that could be construed as a potential conflict of interest.

## Publisher’s Note

All claims expressed in this article are solely those of the authors and do not necessarily represent those of their affiliated organizations, or those of the publisher, the editors and the reviewers. Any product that may be evaluated in this article, or claim that may be made by its manufacturer, is not guaranteed or endorsed by the publisher.

## Footnotes

1
https://covid19.who.int/

2
https://www.ema.europa.eu/
