# Supplementary material for: In vitro Evaluation of Antiviral Efficacy of a Standardized Hydroalcoholic Extract of Poplar Type Propolis Against SARS-CoV-2
Source: Front Microbiol. 2022 Mar 8;13:799546. doi: 10.3389/fmicb.2022.799546 (PMC8958028; doi:10.3389/fmicb.2022.799546)
Supplement: Supplementary file 1 [file Data_Sheet_1.DOCX]

Supplementary Material

# Supplementary Figures

**A**

**E**


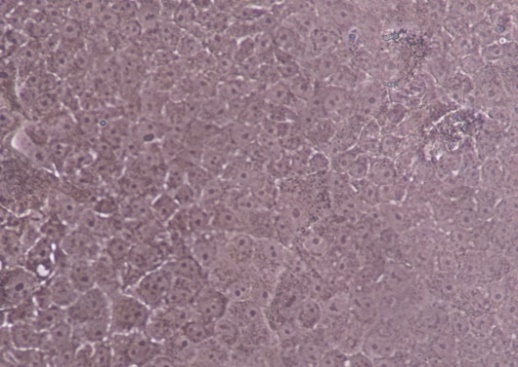

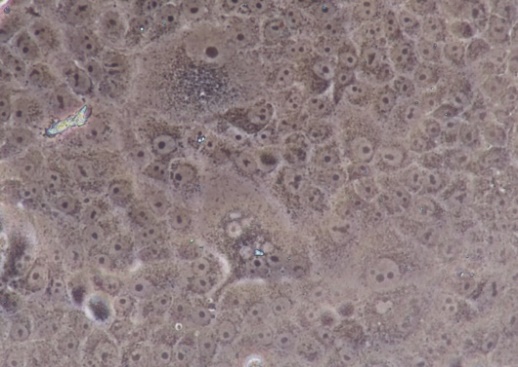

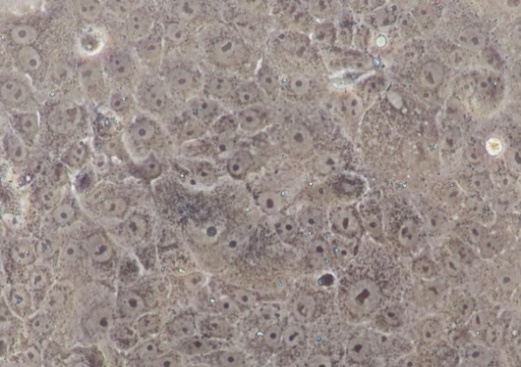

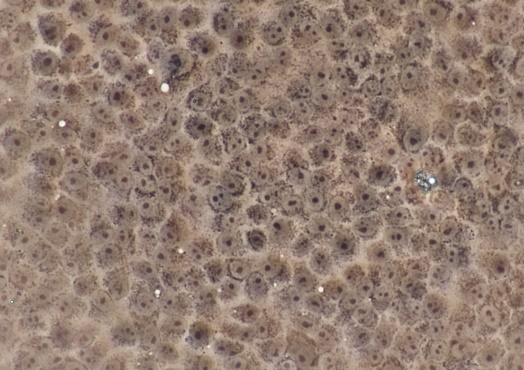

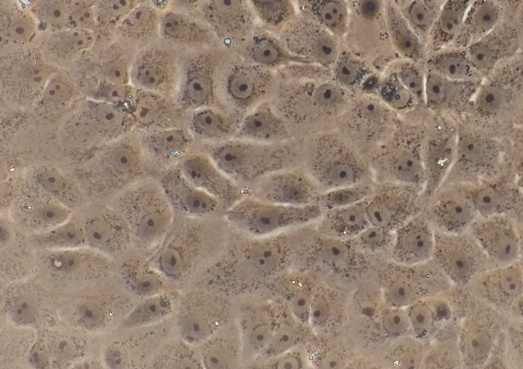

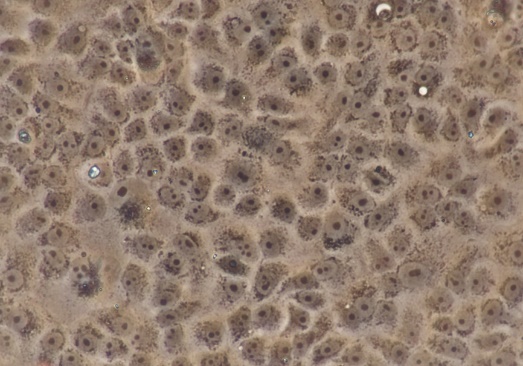


**B**

**F**

**C**

**G**

**D**

**H**

20x

20x

20x

20x

20x

20x

**VERO E6**

**CALU 3**

**Supplementary Figure 1.** **Cell Cytotoxicity 50% (CC_50_) values of sHEP measured in VERO E6 and CALU3 cell lines**. Cell viability of VERO E6 and CALU3 cell lines was measured after addition of different concentrations of sHEP (400 – 200 – 100 – 50 – 25 – 12.5 – 6 – 3 – 1 µg/mL) in the culture medium after 2h, 24h, 48h and 72h p.i. by CellTiterGlo^®^ Luminescent Cell Viability Assay. Panel **(A)** show VERO E6 CC_50_ values at 2h, 24h, 48h and 72h p.i.: 189, 165, 99 and 83 µg/mL of sHEP, respectively. Panel **(E)** show CALU3 CC_50_ values at 2h, 24h, 48h and 72h p.i.: 181, 164, 113 and 55 µg/mL of sHEP, respectively. Panels **(B)**, **(C)**, **(D)** and **(F)**, **(G)**, **(H)** show light microscopy images of VERO E6 and CALU3, respectively, cultured in presence of 12.5 µg/mL or 25 µg/mL of sHEP for 48h. No sign of morphological alterations were noticeable, with the exception of CALU3 showing a trend to acquire triangular-like and elongated shape, yet without visible cytotoxic effects **(H)**.

**Supplementary Figure 2. Effects of sHEP on SARS-CoV-2 entry into the VERO E6 and CALU3 cell lines**. The two cell lines were pre-incubated for 1 hour with 12.5 and 25 µg/mL sHEP, inoculated with the virus and, after two washes, cultured in the presence of sHEP at the same dose used in the pre-incubation. **(A)**, **(E)** Cell viability of VERO E6 and CALU3 cell lines was measured by CellTiterGlo^®^ Luminescent Cell Viability Assay, respectively. **(B)**, **(F)** Ct values refer to negative sense RNA transcripts for N gene in VERO E6 and CALU3 cell extracts, respectively. **(C)**, **(G)** Ct values of E gene RNA detected in VERO E6 and CALU3 cell extracts, respectively. **(D)**, **(H)** SARS-CoV-2 titration (Log TCID_50_/mL) measured in supernatants of VERO E6 and CALU3, respectively. Data points represent the mean (±SD) of three independent experiments. The asterisks indicate statistically significant differences determined by Student’s t test (****, *p*<0.0001; ***, *p*<0.001; **, *p*<0.01; *, *p*<0.05; no asterisk, *p*≥0.05) between infected cells and those infected and pre-treated and cultured in presence of increasing concentrations of sHEP.

**Supplementary Figure 3. Effects of sHEP in VERO E6 and CALU3 infected with SARS-CoV-2 INMI1 at MOI 0.01.** Panels **(A)** and **(C)**: cell viability (%) of VERO E6 and CALU3 measured with CellTiter-Glo^®^ Luminescent Cell Viability Assay at indicated time points. The asterisks indicate statistically significant differences determined by Student’s t test (****, *p*<0.0001; ***, *p*<0.001; **, *p*<0.01; *, *p*<0.05; no asterisk, *p*≥0.05) between infected cells and those infected and cultured in presence of increasing concentrations of sHEP. Data points represent the mean (±SD) of four independent experiments. Panel **(A)**: statistical significance between treated and untreated VERO E6 at 24h was ** for 50 µg/mL; at 48h **** for 50 µg/mL and 25 µg/mL and * for 12.5 µg/mL of sHEP; at 72h only 50 µg/mL had **** of *p*-value. Panel **(C)**: statistical significance between treated and untreated CALU 3 at 24h was ** for 50 µg/mL of sHEP; at 48h *** for 25 µg/mL and ** for 50 µg/mL of sHEP; at 72h only 50 µg/mL had **** of *p*-value. Panels **(B)** and **(D)** shown Ct values of ORF1ab gene measured in SN of both cell lines at indicated time points. Analysis was performed on a pool of SN from four independent experiments.
